# Group ICA of wide-field calcium imaging data reveals the retrosplenial cortex as a major contributor to cortical activity during anesthesia

**DOI:** 10.3389/fncel.2024.1258793

**Published:** 2024-05-10

**Authors:** Alessandro Scaglione, Francesco Resta, Francesco Goretti, Francesco S. Pavone

**Affiliations:** ^1^Department of Physics and Astronomy, University of Florence, Florence, Italy; ^2^European Laboratory for Non-Linear Spectroscopy (LENS), Florence, Italy; ^3^National Institute of Optics, National Research Council (INO-CNR), Sesto Fiorentino, Italy

**Keywords:** ICA decomposition, isoflurane anesthesia, RCaMP, PHP.eB, cerebral cortex, brain states

## Abstract

Large-scale cortical dynamics play a crucial role in many cognitive functions such as goal-directed behaviors, motor learning and sensory processing. It is well established that brain states including wakefulness, sleep, and anesthesia modulate neuronal firing and synchronization both within and across different brain regions. However, how the brain state affects cortical activity at the mesoscale level is less understood. This work aimed to identify the cortical regions engaged in different brain states. To this end, we employed group ICA (Independent Component Analysis) to wide-field imaging recordings of cortical activity in mice during different anesthesia levels and the awake state. Thanks to this approach we identified independent components (ICs) representing elements of the cortical networks that are common across subjects under decreasing levels of anesthesia toward the awake state. We found that ICs related to the retrosplenial cortices exhibited a pronounced dependence on brain state, being most prevalent in deeper anesthesia levels and diminishing during the transition to the awake state. Analyzing the occurrence of the ICs we found that activity in deeper anesthesia states was characterized by a strong correlation between the retrosplenial components and this correlation decreases when transitioning toward wakefulness. Overall these results indicate that during deeper anesthesia states coactivation of the posterior-medial cortices is predominant over other connectivity patterns, whereas a richer repertoire of dynamics is expressed in lighter anesthesia levels and the awake state.

## Introduction

Cortical activity undergoes local and long-range dynamic changes which span across the temporal and spatial dimensions. In particular dynamics at the mesoscale level have been demonstrated to support cognition (Miller and Wilson, 2008; Buonomano and Maass, 2009), sensory processing (Mohajerani et al., 2013), motor learning (Chen et al., 2017), and goal-directed behaviors (Allen et al., 2017). Notably, it has been established that the activity of large-scale networks exhibits extensive similarities between rodents and humans, confirming that multimodal studies conducted in animal models can significantly contribute to our understanding of brain mechanisms in humans (Xu et al., 2022).

Brain states, including wakefulness, sleep, and anesthesia, are characterized by distinct patterns of neural activity, reflecting changes in overall neuronal firing and synchronization. Research has demonstrated that brain state significantly influences the spatiotemporal dynamics of neuronal activity both within and across different brain regions (Pais-Roldán et al., 2020; Meyer-Baese et al., 2022) as well as the evolution of the cortical activity evoked by incoming sensory stimuli (Fanselow and Nicolelis, 1999; Pachitariu et al., 2015; Schwalm et al., 2017). Anesthesia is a powerful tool that allows for systematic control of the brain states (Aedo-Jury et al., 2020). A significant body of evidence suggests that the unconscious state during anesthesia primarily stems from the disruption of long-range connections between cortical areas, particularly in top-down feedback pathways, leading to impaired integration of cortical information (Alkire, 2008; Redinbaugh et al., 2020). Despite the various aspects that have already been described, a comprehensive topographical characterization of cortical network activity in different brain states has not yet been fully analyzed.

The aim of this work was to determine if specific cortical regions might be particularly relevant for sustaining the cortical activity in distinct brain states as modulated by isoflurane anesthesia. To this aim, we applied the group Independent Component Analysis (group ICA) to wide-field cortical imaging recordings. Wide-field imaging has been used to study brain states and slow-oscillation propagation across the entire dorsal cortical mantle (Mitra et al., 2018; Rosenthal et al., 2021; Gutzen et al., 2024).

Group ICA is a tool to unravel brain networks in fMRI recordings both in humans (Calhoun et al., 2001, 2009) and animal models (Whitesell et al., 2021) and it has been recently applied to mice fMRI data to study the Default Mode Network (Whitesell et al., 2021).

Thanks to this approach we identified independent components (ICs) representing elements of the cortical networks that are common across subjects in different anesthesia levels and awake states. We found that ICs related to the retrosplenial cortices coherently presented a marked brain state dependency. Indeed, compared to other cortical regions (somatomotor, somatosensory, visual, posterior parietal), retrosplenial ICs were the most represented during deeper anesthesia and the lower in the awake state, where sensory and motor areas were more prominent. Analyzing the co-occurrence of the ICs we found that activity in deeper anesthesia states was characterized by a strong correlation between the retrosplenial cortices and this correlation decreased toward lighter anesthesia levels and the awake state, where we found a lower correlation between different components.

Overall these results indicate that during deeper anesthesia states coactivation of the retrosplenial cortices is predominant over other connectivity patterns, whereas a richer repertoire of activation dynamics characterized the lighter anesthesia levels and the awake state.

## Results

In this study, isoflurane anesthesia was employed to gradually modulate the brain state from deep anesthesia to wakefulness. Simultaneously, wide-field calcium imaging was performed to monitor neuronal activity in head-fixed mice expressing the red-shifted calcium indicator jRCaMP1b (Montagni et al., 2018; Dana et al., 2019; Resta et al., 2022). Red-shifted GECIs, such as RCaMPs, offer numerous advantages, including increased maximal imaging depth, reduced photodamage, and minimal hemodynamic contribution (Dana et al., 2016; Azimi et al., 2020; Esmaeili et al., 2021; Resta et al., 2022).

**Figure 1:**
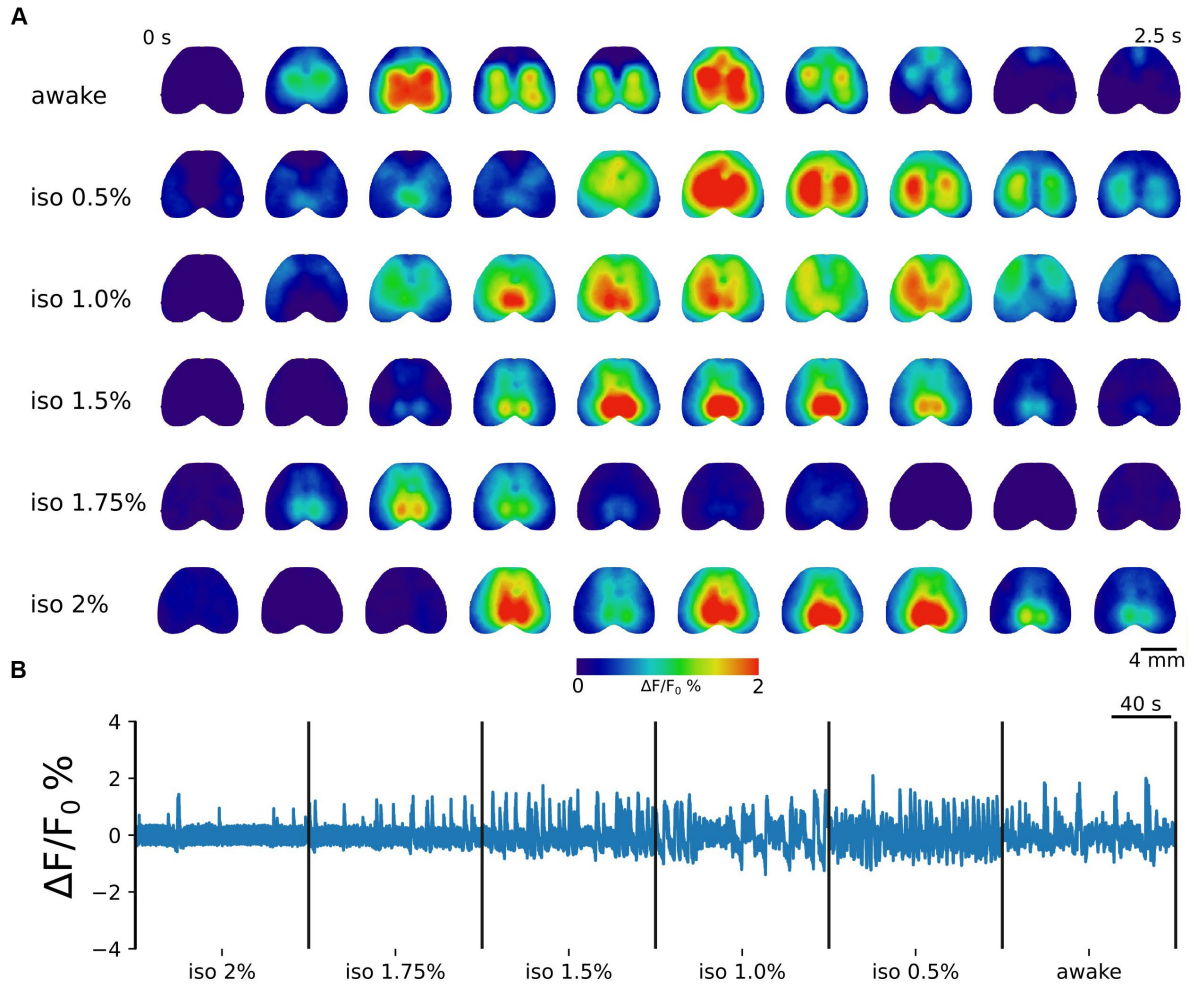
Brain states are associated with distinct cortical dynamics. **(A)** Representative image sequences illustrating cortical activation in the awake state and during increasing levels of anesthesia. The frame time window is 0.25 s. Scale bar = 4 mm. **(B)** Example concatenated trace showing the average cortical activity from 2% isoflurane anesthesia toward wakefulness. The global cortical activity gradually changes from a bistable (up and down-state) to an oscillatory regime in lighter anesthesia levels. A complex distributed activity characterizes the awake state.

To ensure the expression of jRCaMP1b throughout the entire cortex, we utilized AAV-PHP.eB viral vectors (Chan et al., 2017), achieving homogeneous transfection across the dorsal cortical mantle (Supplementary Figure S1). Consistent with prior research (Alkire et al., 2008; Torao-Angosto et al., 2021; Tort-Colet et al., 2023), different anesthesia levels induced distinct patterns of neuronal activity (Figure 1A). Specifically, during deep anesthesia states, we observed the presence of up and down-states, indicating the burst suppression regime, with predominantly global and synchronized activity. In contrast, intermediate and light anesthesia levels displayed oscillatory profiles. Furthermore, the awake state was characterized by fragmented and distributed dynamics (Figure 1B).

Our objective was to characterize how cortical activity organizes itself during different brain states in an unbiased manner. To achieve this, group ICA was used to identify the ICs that decompose cortical activity across subjects and sessions (Figure 1A). In total, 24 ICs were identified across all brain states. Of these components, 21 ICs were retained for further analyses (Supplementary Figure S2), while three were excluded due to their representation of major cortical vessels or the presence of a distinct noisy pattern.

### Group ICA identifies ICs that are common across subjects and brain states

**Figure 2:**
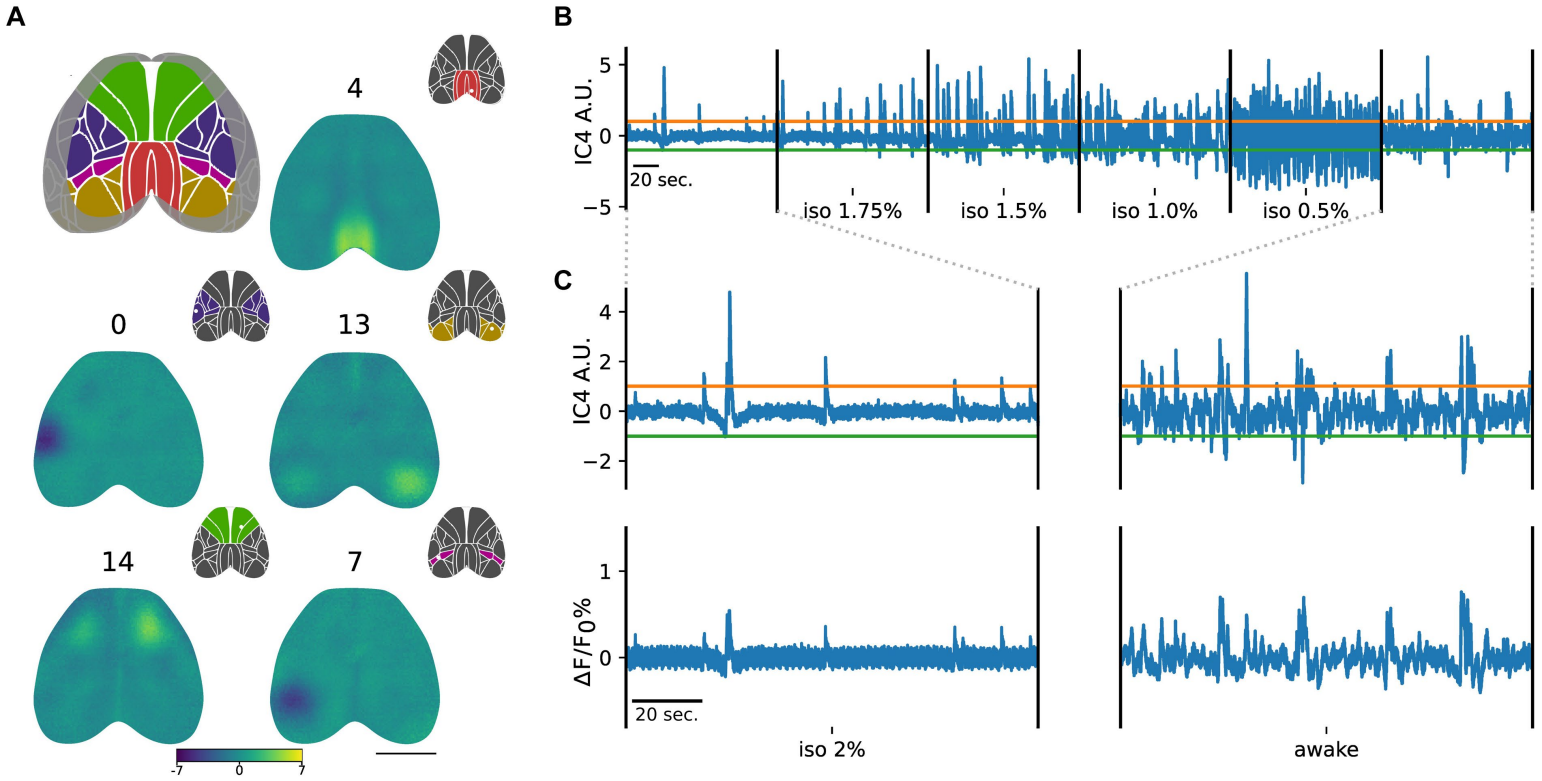
Group ICA identifies ICs that are common across subjects and brain states. **(A)** ICs are categorized into cortical groups in somatosensory (blue), somatomotor (green), retrosplenial (red), posterior parietal (purple), and visual (dark yellow), based on the Allen Brain Atlas. Scale bar = 3 mm. Representative ICs for each group are depicted with corresponding color codes. Insets indicate the assigned group based on the IC maximum (white dot). **(B)** Example of the time course of IC4 (retrosplenial group) across different levels of anesthesia and wakefulness. Green and orange lines represent the positive (mean + 3 standard deviations of the iso 2% state) and negative (mean - 3 standard deviations of the iso 2% state) thresholds, respectively. **(C)** Magnified view of the IC4 time course in iso 2% and awake states aligned with their respective global average activities. Remarkably, deep anesthesia exhibits a strong similarity between the IC4 time course and the average global activity, while this relationship weakens during the awake state.

Group ICA of the imaging dataset from three subjects in different brain states yielded independent components (ICs) confined to various regions of the mouse cortex. The ICs were registered on the Allen Brain Atlas (CCFv3) and tagged within the visual, somatosensory, retrosplenial, posterior parietal, and motor groups, based on the specific area, in the atlas, where the maximum value of each component was located (Figure 2A).

Using this approach, we identified 10 somatosensory ICs, 3 somatomotor ICs, 3 retrosplenial ICs, 2 posterior parietal ICs, and 3 visual ICs (Supplementary Figure S2). The reproducibility of the components across sessions of individual animals has been verified by measuring the correlation coefficients between the back reconstructed ICs extracted with group ICA and the ICs extracted by applying ICA separately to each session of each animal. The results of the comparison are presented in Supplementary Figure S3, showing a remarkable high agreement between the two sets of components on a session by session basis (Supplementary Figure S3A).

In addition to providing spatial maps, group ICA also generates time courses for each component (Figure 2B). In Figure 2, the time course of IC_4_ in a representative subject is displayed, aligned with the global cortical activity during deep anesthesia and the awake state (Figure 2C).

To determine the contribution of each component to the cortical activity, we applied a threshold to the ICs’ time courses in the deeper anesthesia condition. We used this state of global inactivation as a reference point to identify significant activation of each component, represented by crossing the threshold (see methods). We observed that the number of points crossing the threshold was modulated by the level of anesthesia. Specifically, as the level of anesthesia decreased, there was an increase in overall neuronal activation, resulting in a drastic increase in the number of crossings for all ICs (Figure 2B).

Overall these results suggest that group ICA can be successfully applied to wide-field calcium imaging data yielding components that are stable across sessions of different animals and can be used to decompose cortical activity into several ICs whose time course is coupled to the global cortical activity.

### Brain states are characterized by different sets of ICs

Since each component showed an increased number of threshold crossings with decreasing levels of anesthesia, we aimed to investigate how the relative occurrence of each IC is modulated by the brain state. To achieve this, in each state, we examined the relative proportion of occurrences of each IC and compared it with the expected number of occurrences when considering either the deepest state of anesthesia or the awake state.

**Figure 3:**
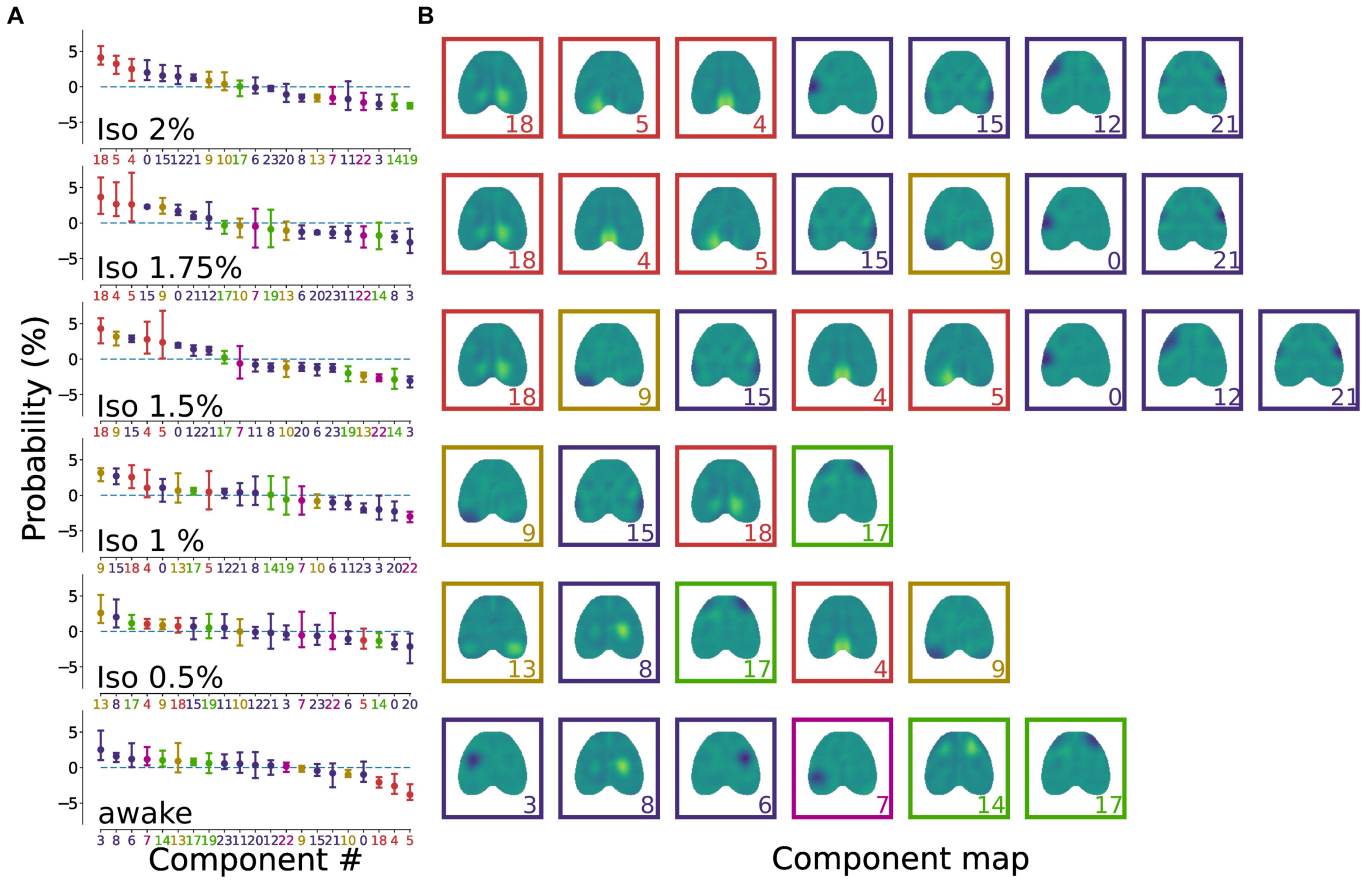
Brain states are characterized by a distinct distribution of ICs. **(A)** Dot plots representing the state-specific distributions showing the normalized relative occurrence of ICs in different brain states. The dots represent the mean and the bars represent the 95% CI. The sorted (descending) probability distribution of all brain states displays a prominent negative slope, indicating a non-homogeneous distribution of IC probabilities. The colors of the dots, bars and component number on the x-axis match the coloring for the cortical groups reported in Figure 2A. **(B)** Representation of the ICs that exceed the expected occurrence for each brain state. Deeper anesthesia states are characterized by retrosplenial and somatosensory components, while lighter anesthesia and wakefulness display a richer repertoire of component groups. Colors of the boxes match the colors for the cortical groups reported in Figure 2A.

When arranging the components in descending order based on their relative frequency of occurrence (Figure 3A), we observed that each state was characterized by a distinct set of components whose relative occurrence surpassed the expected frequency (Figure 3B). In addition, the sorted probability distribution of all brain states exhibited a negative slope, indicating an uneven distribution of IC probabilities, implying that certain groups of ICs were more prevalent than others (Figure 3A).

The results revealed that lighter levels of anesthesia (iso 1–0.5%) exhibited a reduced number of prominent components compared to deeper anesthesia (iso 2–1.5%) and wakefulness. This finding suggests a more balanced contribution of the ICs during the oscillatory state. Moreover, deeper anesthesia states were denoted by the prevalence of retrosplenial and somatosensory components, while lighter anesthesia and wakefulness displayed a broader repertoire of component groups.

### ICs of the retrosplenial group are prevalent during deep anesthesia

**Figure 4:**
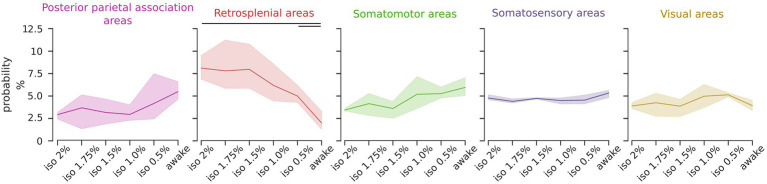
The relative representation of the retrosplenial group is modulated by the brain state. Line plots depict the relative occurrence of each IC group across brain states. Solid lines represent the mean, and the shadows represent the 95% CI. The occurrence of the retrosplenial group exhibits a significant dependence on the brain state (RM ANOVA *p* < 0.001, *post hoc* pairwise *t*-test with holm correction *p* < 0.05) while the other groups are not significantly affected by the brain states.

The uneven distribution of ICs probabilities arises from anesthesia-induced modulation of their relative occurrence. To assess which component class was modulated by the level of anesthesia we analyzed the proportional occurrence of each class from deep anesthesia to wakefulness. The findings revealed that the retrosplenial group exhibited a statistically significant brain state dependency [RM ANOVA *F* (5, 10) = 19.46, *p* < 0.001], whereas other classes showed no significant trends or a uniform distribution (Figure 4). A *post hoc* analysis revealed that the probability of occurrence of the retrosplenial group was significantly higher in the deepest level of anesthesia compared to the awake state [pairwise repeated *t*-test with holm correction, *t* (2) = 21.04, *p* = 0.03]. These results were also tested with respect to the total number of ICs selected for the group ICA analysis. Specifically, we repeated the same analysis selecting 16, 20, 24, 28, and 32 ICs. Supplementary Figure S5A shows that the result obtained using 24 ICs is preserved over the chosen range of ICs numbers supporting the stability of the result with respect to moderate changes in the chosen number of ICs.

These findings suggest that the relative contribution of the retrosplenial cortices to the overall activation can serve as a predictor of the brain state.

### Retrosplenial ICs correlation decrease from deep anesthesia to wakefulness

**Figure 5:**
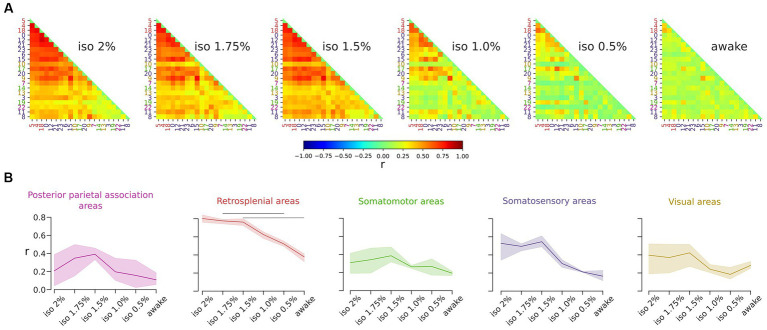
Correlations among ICs of the retrosplenial group decrease during the transition from deep anesthesia to the awake state. **(A)** Correlation matrices display the r value for all pairs of ICs. Global correlations decrease as anesthesia levels decrease. **(B)** Line plots illustrate the level of correlation between ICs within each group across brain states. Solid lines represent the mean, and the shadows represent the 95% CI (Confidence Interval). The retrosplenial ICs exhibit the highest correlation value, which significantly depends on the brain state (RM ANOVA *p* < 0.001, *post hoc* pairwise *t*-test with holm correction *p* < 0.05).

Our results showed that the ICs in the retrosplenial group exhibited a pronounced dependency on brain states, being prominent in anesthetized states and proportionally infrequent during wakefulness. To further investigate the role of the retrosplenial cortex, we measured the correlation among the time courses of ICs across states (Figure 5). Our findings indicated a decrease in overall correlation with diminishing levels of anesthesia. In addition, we observed the highest correlation values between ICs of the retrosplenial group during the deepest state of anesthesia (r = 0.80). These correlations significantly decreased during the awake states (r = 0.38) [RM ANOVA *F* (5, 10) = 39.4, *p* < 0.001] (Figure 5B). Finally, to ensure the robustness of our findings, we assessed the stability of the result with respect to a moderate variation in the number of ICs. Supplementary Figure S5B shows that the result obtained using 24 ICs is preserved over the chosen range of ICs numbers, supporting the stability of the result with respect to variation in the selected number of ICs.

These results show that brain states directly modulate the correlations among the time course of the ICs especially in the retrosplenial cortices.

## Discussion

In this study, we used isoflurane anesthesia to systematically manipulate the brain state from deep anesthesia to wakefulness. Employing the group ICA on wide-field calcium imaging datasets we collected evidence indicating that neuronal activity in the retrosplenial cortex is significantly modulated by the brain states.

Wide-field calcium imaging data are inherently multidimensional; they represent the temporal evolution of the calcium indicator fluorescence for each single pixel in the field of view. Because of this, several authors have been employing data dimensionality reduction algorithms for computation purposes identifying groups of pixels that functionally behave similarly (Makino et al., 2017; Musall et al., 2019; Saxena et al., 2020; Cecchini et al., 2021; Peters et al., 2021; Quarta et al., 2022; West et al., 2022; Cramer et al., 2023; Nietz et al., 2023). This leads to dynamic segmentations or cortical parcellations that depend on experimental manipulation (Nietz et al., 2022) as opposed to static parcellations based on cytoarchitectonic features of the cerebral cortex (Wang et al., 2020).

Algorithms based on principal components analysis (PCA) or singular value decomposition (SVD) require spatial components to be orthogonal with each other; this represents a stringent assumption to be verified in biological data that leads to the mixing of the underlying biological signals (Brown et al., 2001; Roberts and Everson, 2001). For this reason, authors that have employed this method on wide-field calcium imaging data have done so for data reduction purposes and consequently to reduce computational cost (Musall et al., 2019; Peters et al., 2021), or to extract general features of the data, such as the direction propagation of the neural activity (Cecchini et al., 2021). To avoid the orthogonality assumption, several authors have been using other methods that require less stringent assumptions such as non negative matrix factorization (NMF) or ICA.

NMF assumes that the data are non-negative, this means that it cannot be directly applied to wide-field calcium imaging data as the ∆F/F_o_ can be either positive or negative and some pre-processing is needed to make the signal completely positive (MacDowell and Buschman, 2020; Quarta et al., 2022). Alternatively, negative values can be used by allowing the component time course to take negative values but constraining the spatial components to the Allen CCF Brain Atlas (Saxena et al., 2020; Cramer et al., 2023).

ICA has been used before to find functional groups of pixels that can decompose the fluorescence signal (Makino et al., 2017; West et al., 2022; Nietz et al., 2023), and it has been used to identify dynamic parcels of the cerebral cortex that change across multiple timescales and behavioral states of animals. In fact, in a recent work Nietz et al. (2023) have shown that only some components are preserved across different timescales and animal behaviors some others are not and display only transient activation which is timescale and behavior specific and lost when observing long time periods. Supporting the view of a core stable network that is reconfigured for specific processing by the transient activated networks. To show this, the authors cleverly compared the full dataset to partitions of itself to identify similar or different ICs and compare the results across animals. In this work, instead, we are interested only in the effect of the behavior on the stable networks that are similar across animals, for this reason, we employed group ICA that avoids the problem of the variability of components among subjects (Nietz et al., 2023), a problem that is also present for both PCA and NMF approaches. In fact, group ICA extracts stable components across subjects thanks to the data reduction steps before the ICA decomposition stage (Calhoun et al., 2001) without the need for any additional assumption. In addition, by knowing that during the prolonged down states in the deepest state of anesthesia, neuronal activity was minimal (Alkire et al., 2008; Brown et al., 2010), we used this as a reference to construct the thresholds for each component time course. This allowed us to identify the data points where components significantly contributed to the overall fluorescence signal.

The application of group ICA to wide-field imaging data allowed us to discover 21 ICs that, with their respective time courses, decompose neural activity of the entire dorsal portion of the cortex. Interestingly, there is a remarkable similarity between the functional map of each ICs and the structural map of the Allen Brain Atlas. In addition, when considering the time courses of the ICs, we observed a decrease in overall correlation when transitioning from the anesthetized to the awake state. This decrease in correlation is well in agreement with the observation that anesthesia induces synchronous activation at a multi-scale level (Bharioke et al., 2022). Taken together, this evidence supports the application of group ICA to wide-field imaging data.

Although we employed group ICA to wide-field calcium imaging data from headfixed mice, there could be issues in applying group ICA to imaging data collected in dynamic or complex contexts due to potential motion artifacts. For instance, when conducting complex social interaction experiments using miniscopes in freely moving animals, it’s crucial to carefully assess motion artifacts arising from rapid and large movements. In such scenarios, the first step should be to assess the effect of movement artifacts on the ICs. One approach is to carefully analyze both the map and time course of each IC while performing kinematic measurements on the animals for verification. If motion artifacts are similar across sessions and animals, even if they occur at different times, group ICA should be able to capture those artifacts in some specific components. Therefore, removing the movement-related ICs could be enough. In the case in which motion artifacts vary greatly among sessions or animals, one possible solution could involve employing motion correction algorithms (Guizar-Sicairos et al., 2008; West et al., 2022).

Analyzing the occurrence of each IC relative to the different levels of anesthesia, we demonstrated distinct patterns. The burst suppression regime (iso 2–1.5%) was primarily characterized by the prevalence of the retrosplenial and somatosensory groups (Figure 3). Additionally, the oscillatory states (iso 1–0.5%) exhibited a balanced contribution from numerous components across different groups. Notably, the awake state displayed an uneven distribution comprising various groups, with the retrosplenial group showing the lowest occurrence. Furthermore, exploring the impact of brain states on the occurrence of different groups confirmed that only the retrosplenial group exhibited significant brain state-dependent modulation. Specifically, as the anesthesia level decreased, the occurrence of the retrosplenial group decreased significantly (Figure 4). It should be noted that a significant portion of neuronal activity in the brain is associated with movement execution (Stringer et al., 2019) accounting for the movement itself and/or changes in the movement-related internal state (Musall et al., 2019). Since movement-related activity is widespread throughout the brain, there is a risk of attributing this activity to other processes such as cognitive or sensory integration (Syeda et al., 2024) especially if behavior is not properly monitored. Here, we considered movement-related cortical activations as part of the spontaneous behaviors expressed during wakefulness and in part during light anesthesia, without associating ICs to specific cortical functions. Indeed, while it is very likely that neural activations captured by the group ICA ICs are coupled with the movement execution during the awake state, it is important to note that such components are also present during the deep stages of anesthesia when animals do not show any movement activity. This suggests that the changes in the relative proportion of ICs and the correlation among them are mainly due to the different levels of anesthesia. Further implementation of experimental and analytical methods to disentangle the contribution of movements from the other computations performed by the cortex could give additional insights into the brain state-specific cortical dynamics.

Although we cannot assess synchronicity among spikes of cortical neurons, the result in Figure 5 shows that cortical areas tend to coactivate during deeper anesthesia states which is compatible with the previously reported synchronization of spiking activity among large portions of the cortex (Lee et al., 2021). The lower correlation values observed in awake (Figure 5) could indicate variations in behavior within the same state, such as transient attentional loading, movement planning and execution, and active versus quiet wakefulness. In addition, our findings suggest a crucial involvement of the retrosplenial cortices in unconscious brain states. Specifically, the repertoire of oscillatory activity in deeper levels of anesthesia is characterized by recurring participation of the retrosplenial cortices in the overall cortical activation. These results are in line with previous evidence suggesting that the retrosplenial cortex may play a role in the recovery of consciousness in patients with vegetative or minimally conscious states (Laureys and Schiff, 2012).

The retrosplenial region, which encompasses a significant portion of the posterior medial surface of the cortex in rodents (Alexander et al., 2023; Foster et al., 2023), is known for its high level of integration within the medial cortical subnetwork. It receives input from the claustrum and the hippocampus and exhibits direct connections with various sensory and high-order associative areas, including the medial frontal cortex (Zingg et al., 2014). Functionally, the retrosplenial cortex has been linked to crucial cognitive processes such as learning, memory, and navigation (Todd et al., 2019) and it is a central hub of the DMN (Whitesell et al., 2021). The role of the retrosplenial cortex has been also explored in the context of dissociative behavioral states induced by subanesthetic doses of ketamine in rodents (Vesuna et al., 2020). The authors found that specific oscillatory activity (1–3 Hz) emerges selectively in the retrosplenial cortex upon administration of dissociative drugs. Interestingly, a pronounced pattern of gamma-burst activity has recently been observed in the bilateral posteromedial cortex during ketamine anesthesia (Arena et al., 2022). Taken together, this evidence, along with our findings, indicates that modulation of retrosplenial cortex activity could be associated with the disruption of widespread integration of complex cortical interactions, which may directly contribute to the breakdown of the properties necessary for sustaining consciousness.

In this work, using group ICA we have gathered evidence suggesting brain state-dependent activation of the retrosplenial cortices. Future research could further explore a potential role for this region in supporting consciousness by causally modulating the retrosplenial activity, such as using opto- or chemo-genetics. It is worth noting that in this study, we investigated cortical activation on a large scale using an analysis method commonly employed in clinics. These two points are important aspects for translating research findings from rodents to humans.

## Methods

### Experimental procedures

All experiments conducted in this study adhered to the guidelines set forth by the Italian Minister of Health (authorization number 857/2021). Three C57BL/6 J adult mice (8–10 months old) of both sexes were utilized. The mice were housed in a controlled environment with regulated temperature and humidity and provided with unrestricted access to food and water.

### Intact-skull window and virus injection

Mice were anesthetized with isoflurane (3% for induction, 1–2% for maintenance) and placed in a stereotaxic apparatus (KOPF, model 1900) as previously described (Resta et al., 2022). Ophthalmic gel (Lacrilube) was applied to prevent eye drying, and body temperature was maintained at 36°C using a heating pad. Lidocaine 2% was used as a local anesthetic. The skin and periosteum were cleaned and removed, and bregma was marked with a black fine-tip pen. Transparent dental cement (Super Bond C&B – Sun Medical) was then applied to cover the entire exposed skull surface and to glue a custom-made aluminum head-bar, placed behind lambda. To achieve widespread expression of jRCaMP1b over both hemispheres, the viral construct ssAAV-PHP.eB/2-hSyn1-NES_jRCaMP1b-WPRE-SV40p(A) (Viral Vector Facility, Zurich, Switzerland) was systemically injected into the retro-orbital sinus. The virus titer was 1.5 × 10^13^ vg/ml and a total volume of 150 μL was injected.

After the surgery, mice were recovered in a temperature-and humidity-controlled room, with food and water *ad libitum* for 3 weeks before recordings. The intact skull cranial window was implanted 1 month after injection and imaging sessions started after 1 week after the window implantation to allow full recovery after the surgical operation. On day 7 post-surgery, mice were acclimated to the setup and habituated to being handled (20-min habituation session). In the subsequent week, mice were habituated to resting with their heads fixed under the microscope in a dark environment with white noise (two sessions of 20 min each).

### Wide-field imaging

Wide-field imaging was performed in a dark room using a custom-made microscope. The excitation source for jRCaMP1b was a red-light beam emitted by diodes (595 nm LED light, M595L3 Thorlabs, New Jersey, United States), and the excitation band was selected using a bandpass filter (578/21 nm, Semrock, Rochester, New York, United States). The light beam was directed to the objective (TL2X-SAP, Thorlabs, New Jersey, United States) through a dichroic mirror (606 nm, Semrock, Rochester, New York, United States). The fluorescence signal of jRCaMP1b was collected through a band-pass filter (630/69, Semrock, Rochester, New York, United States) and focused onto the sensor of a high-speed complementary metal-oxide semiconductor (CMOS) camera (Orca Flash 4.0 Hamamatsu Photonics, NJ, United States) using a tube lens (500 nm). The camera captured images at a resolution of 512 × 512 pixels, covering a square field-of-view of 11.5 mm. The sampling rate was 40 Hz.

We utilized a custom-made platform comprising a square tube to position the animals beneath the microscope objective and secure the metal post that had been implanted on their heads. In this condition, the animals rest in the objective focal plane with no ability to move around or run.

Anesthesia induction was initiated at a concentration of 3–4% isoflurane. Following induction, the animals were transferred to the experimental setup, and the isoflurane concentration was reduced to 2%. Before imaging, anesthesia levels were stabilized, with a waiting period of 10–15 min after any adjustments to the isoflurane concentration. Longer waiting times were required before imaging in the awake state for anesthesia recovery. Each recording session had a duration of 120 min. During the deep and intermediate anesthesia states (isoflurane range 2–1%), the animals did not express any behavior (i.e., detectable movements). In the lighter anesthesia state (isoflurane 0.5%), mice started exhibiting small whisker twitches. When awake, a complete repertoire of facial and limb movements was observed. The mice were head-fixed, and their bodies were constrained within a square-sized framework. Trained mice were habituated to this positioning and did not show large movements.

### Immunohistochemistry and two-photon imaging

Brain slices for histological evaluation were prepared as previously reported (Resta et al., 2022). Briefly, mice were perfused with 0.1 M PBS and 4% paraformaldehyde. Brain slices (100 mm thick) were prepared using a Vibratome (Vibratome Series 1500—Tissue Sectioning System), washed, and incubated with NeuN antibody (1:200, Sigma, ABN78) overnight at 4°C. After washing, slices were treated with a fluorescent Alexa Fluor 514 antibody (1:250, ThermoFisher, A-31558) and mounted on glass slides.

Images were acquired using a two-photon microscope (Thorlabs Bergamo) equipped with a Ti-Sa laser (Coherent Chameleon) tuned to 1,064 nm for jRCaMP1b and 950 nm for NeuN (Alexa Fluor 514 reporter) a × 20/0.95 NA water immersion Olympus objective. Emission filters (Semrock) were 520/35 nm and 630/69 nm for NeuN and jRCaMP1b channels, respectively.

### Brain state modulation

Brain state modulation was achieved by changing isoflurane concentration. Specifically, the experiments began at the deepest level of anesthesia with 2% isoflurane. Then, the anesthetic concentration was gradually reduced to 1.75, 1.5, 1.0, and 0.5%, allowing 10 min for the neuronal activity to stabilize at each concentration. Based on previous work (Song et al., 2018; Dasilva et al., 2021; Torao-Angosto et al., 2021; Tort-Colet et al., 2023), we selected these percentages to study the deep, intermediate and light levels of anesthesia. In fact, high concentrations of isoflurane (2–1.75%) stabilize global rhythmic activity (averaged across the entire field of view) at approximately 0.1–0.2 Hz achieving “deep” anesthesia level. Slight adjustments to the isoflurane concentration (1.5%) result in global activity exhibiting higher upstate frequencies of 0.2–0.4 Hz, categorizing recordings in this state as a medium anesthesia level. Finally, the light anesthesia level is achieved at lower concentrations, with global rhythmic activity showcasing prominent oscillatory dynamics. A complete recovery of characteristic behaviors such as motion, whisking, grooming, and liking was expected before the awake imaging session.

## Data analysis

All analyses were performed using custom-written Python scripts with the exception of the group ICA analysis and the ICASSO analysis which have been carried out using the gift toolbox in MATLAB.

### Image pre-processing

To ensure the consistency of the field of view across sessions we performed offline image registration as previously described in Scaglione et al. (2022). Briefly, each frame of the fluorescence data was first downsampled with a factor of 4 resulting in a pixel size of about 90 μm and then offline registered by aligning each frame to two reference points (corresponding to bregma and lambda) that were previously marked on the dental cement during the surgery procedure. After the image registration across all days, subjects and groups, we kept only the regions in the field of view that were common to all sessions (Figure 2A). The more marginal regions were excluded from the analysis and thus the final area analyzed contains at most a very small portion of the lesion. To be able to compare changes in fluorescence across subjects and sessions, the raw image stacks, collected with the wide-field microscope, were expressed in percent changes (∆F/F_0_) with respect to its mean session fluorescence value (F_0_) for each individual pixel. To remove slow drifts, the ∆F/F_0_ was then high-pass filtered (0.1 Hz) setting the bandwidth of the analyzed fluorescence signal from 0.1 to 20 Hz.

The normalized fluorescence signals from different levels of anesthesia, from iso 2% to awake, were then concatenated together into a single session (Figure 1B). In this way, a total of 6 sessions (2 recordings per animal) constitute the dataset analyzed with group ICA.

### Group ICA and ICs classification

To be able to decompose the fluorescence signal in independent components (ICs) across subjects and sessions, we decided to employ the group ICA analysis, originally developed to make group inferences in the context of fMRI data. The main strength of this method lies in the extension of ICA from a single subject to a group of subjects. This is achieved by utilizing a series of data reduction steps that compresses data in the temporal domain, thereby lowering the computational requirements of ICA for the full dataset. In this way, the ICs found do not rely on any model of the neural dynamics since it uses ICA. Additionally, the same components are already generalized across subjects and sessions. Moreover, using this approach, once the ICs are found through ICA, the spatial representation of the component and its time course for a specific session can be expressed as a series of matrix multiplication steps (Calhoun et al., 2001), the back reconstructed components.

We used the GIFT toolbox^1^ to carry out the group ICA analysis on the 6 recording sessions to extract the group ICA ICs (see Supplementary Table S1 for the list of options and parameters used to run the GIFT toolbox). ICASSO (Himberg et al., 2004) was then used to ensure the reliability of the ICs. In fact, ICASSO runs the ICA algorithm a given number of times by bootstrapping the data and or changing the initialization parameters of ICA between runs. Subsequently, the estimates obtained from each run are grouped based on mutual similarities through agglomerative clustering. A reliable estimate of the ICs is then obtained by using the centrotype of each cluster generated by the different ICA runs.

The number of ICs was selected as the number that minimizes the R-index which provides a measure for the compactness and separation of the clusters found when running ICASSO. In fact, while ICASSO can be used to estimate reliable ICs by selecting the centrotype of the cluster generated by each individual run of the ICA, as pointed out by Li et al. (2007), the compactness of the clustering performed during ICASSO can be used to validate the dimensionality or the order selection of the ICA. In fact, the stability of the ICs is closely related to the order selection. Li et al. (2007) show that by increasing the number of components, the compactness of the clustering deteriorates leading to overlapping clusters or, in the case of very high dimensions, the different Monte Carlo runs lead to IC estimates that do not display significant correlations making it difficult to define reliable clusters of single ICs.

Once the ICs were identified, those components whose maximum activation was mostly confined over the midline and or showed clear vasculature were discarded as the change in fluorescence signal along the midline can be contaminated by hemodynamic or movement artifacts.

To check that ICs extracted with group ICA are present in each session from each subject, we compared the back reconstructed components of each session, IC^gICA^_i_ (*i* = 0…23), with the ICs extracted running ICA on each single session separately, IC^ICA^_j_ (*j* = 0…23), together with ICASSO to extract the most reliable components. We then computed the Pearson correlation coefficient between the two sets of components. Thus, for each session a correlation matrix R is obtained whose elements *r_i,j_* represent the correlation coefficient between the i-th back reconstructed component, IC^gICA^_i,_ with the *j*-th single ICA component, IC^ICA^_j_. A correlation coefficient of 1 is obtained when two components have the same weights up to a multiplicative constant. Finally, columns of each correlation matrix are rearranged according to a rectangular assignment algorithm which associates the component on the back reconstructed ICA to at most one element of the single session ICA components based on the maximum value of the |R|.

Once the global ICs maps were obtained, the location of the pixel with the highest value in the map, independently of the sign, was marked. This location was used to name the component according to the Allen Brain Atlas. Finally, components were grouped into 5 different macro areas (first degree of division of the cerebral cortex according to the Allen Brain Atlas) (Figure 2A): (1) Posterior Parietal Association; (2) Retrosplenial; (3) Somatomotor, (4) Somatosensory, and (5) Visual cortical areas. It is important to note that when we are trying to associate the ICs to the anatomical areas defined by the Allen Brain Atlas that are defined by the cytoarchitectonic organization of the cerebral cortex. We are resourcing to the Allen Brain Atlas to keep the same nomenclature and to group cortical parcels into general macro areas that are well accepted and understood.

### ICs characterization during different stages of anesthesia

To quantify the contribution of the ICs to the overall fluorescence signal during the different stages of anesthesia, the time course of every IC in each session was back-reconstructed through group ICA. To identify timepoints where the component displayed a significant change with respect to its background in the time domain, a threshold was set to three times the standard deviation of the time course of the IC during the deepest state of anesthesia (iso 2%) $th=\pm 3*\sigma_{i}^{iso\;2\%}$ . A binary vector for each IC was obtained by setting 1 every time that the component time course surpassed the threshold and zero otherwise. The sum of each of these binary vectors, N_IC,ST_, was defined as the number of activations and was used as a proxy to assess a significant impact of the IC on the ∆F/F_0_ signal. Therefore, the relative proportion of the number of activation N_IC,ST_/∑_IC_N_IC,ST_ represents the proportion of timepoints that an IC significantly impacted the fluorescence signal compared to all other ICs for that particular state ST. This number equals 1 for state ST, only if the time course of an IC passes its threshold for the considered state and all others do not.

To identify the most prominent components of each state, the relative proportion of activations for each component was compared to the expected proportion of activations. The expected proportion was determined by simulating 10,000 threshold crossings from the deepest state of anesthesia or the awake state. Specifically, we randomly sampled 5,000 threshold crossings with replacement from each state, creating a distribution of expected occurrences free from bias due to differences in crossing numbers between states and independent of the state. Subsequently, we used the expected proportion of activations as a threshold to identify prominent components. Finally, a component was considered prominent if the lower bound of its 95% confidence interval for the relative proportion calculated across subjects (*N* = 3) exceeded its expected proportion obtained through the aforementioned random sampling.

### Correlations among ICs of the same cortical areas

To study how components combined with each other to contribute to the overall fluorescence signal, for each session in each brain state we computed the Pearson correlation coefficient r between the binary vectors of the ICs. Assuming that each such vector has at least a zero and a one, a correlation coefficient of 1 implies that both vectors are the same and their underlying ICs are surpassing their threshold in the same timepoint meaning that both components are being coactivated. By contrast, a correlation coefficient of −1 implies that the two components are passing their respective thresholds in different time points. By computing this correlation between each pair of ICs a correlation matrix R is obtained for each brain state in each session.

## Statistical analyses

To quantify if the relative proportions of the activation for the 5 cortical macro areas were significantly modulated by the state. First, the relative proportion of each single brain region in the macro area was averaged, second, this variable was then used in a Repeated Measure ANOVA, factor STATE with levels: iso 2%, iso 1.75%, iso 1.5%, iso 1.0%, iso 0.5% and awake. Similarly, to quantify the effect of the correlations for the macro areas of the cortex, the Pearson correlations coefficient of each pair of regions in the areas was transformed using the Fisher z-transform and averaged and this variable was then used as the dependent variable for a Repeated measure ANOVA with factor STATE with the same levels of the previous analysis. If there was a significant effect of the main factor on the variable of interest, a *post hoc* analysis using repeated *t*-test was performed on all pairs of states using the Holm correction to account for the family-wise error. In all analysis the sample size corresponds to the number of animals, *N* = 3.

## Data availability statement

The original contributions presented in the study are included in the article/Supplementary material, further inquiries can be directed to the corresponding authors.

## Ethics statement

All experiments conducted in this study adhered to the guidelines set forth by the Italian Minister of Health (authorization number 857/2021). The study was conducted in accordance with the local legislation and institutional requirements.

## Author contributions

AS: Conceptualization, Data curation, Formal analysis, Methodology, Software, Visualization, Writing – original draft, Writing – review & editing. FR: Conceptualization, Data curation, Formal analysis, Investigation, Methodology, Visualization, Writing – original draft, Writing – review & editing. FG: Validation, Writing – review & editing, Formal analysis, Visualization. FP: Conceptualization, Funding acquisition, Project administration, Resources, Validation, Writing – review & editing.

## Footnotes

^1^ https://www.nitrc.org/projects/gift/
