# Supplementary figures and images for: Group ICA of wide-field calcium imaging data reveals the retrosplenial cortex as a major contributor to cortical activity during anesthesia
Source: Front Cell Neurosci. 2024 May 10;18:1258793. doi: 10.3389/fncel.2024.1258793 (PMC11116703; doi:10.3389/fncel.2024.1258793)

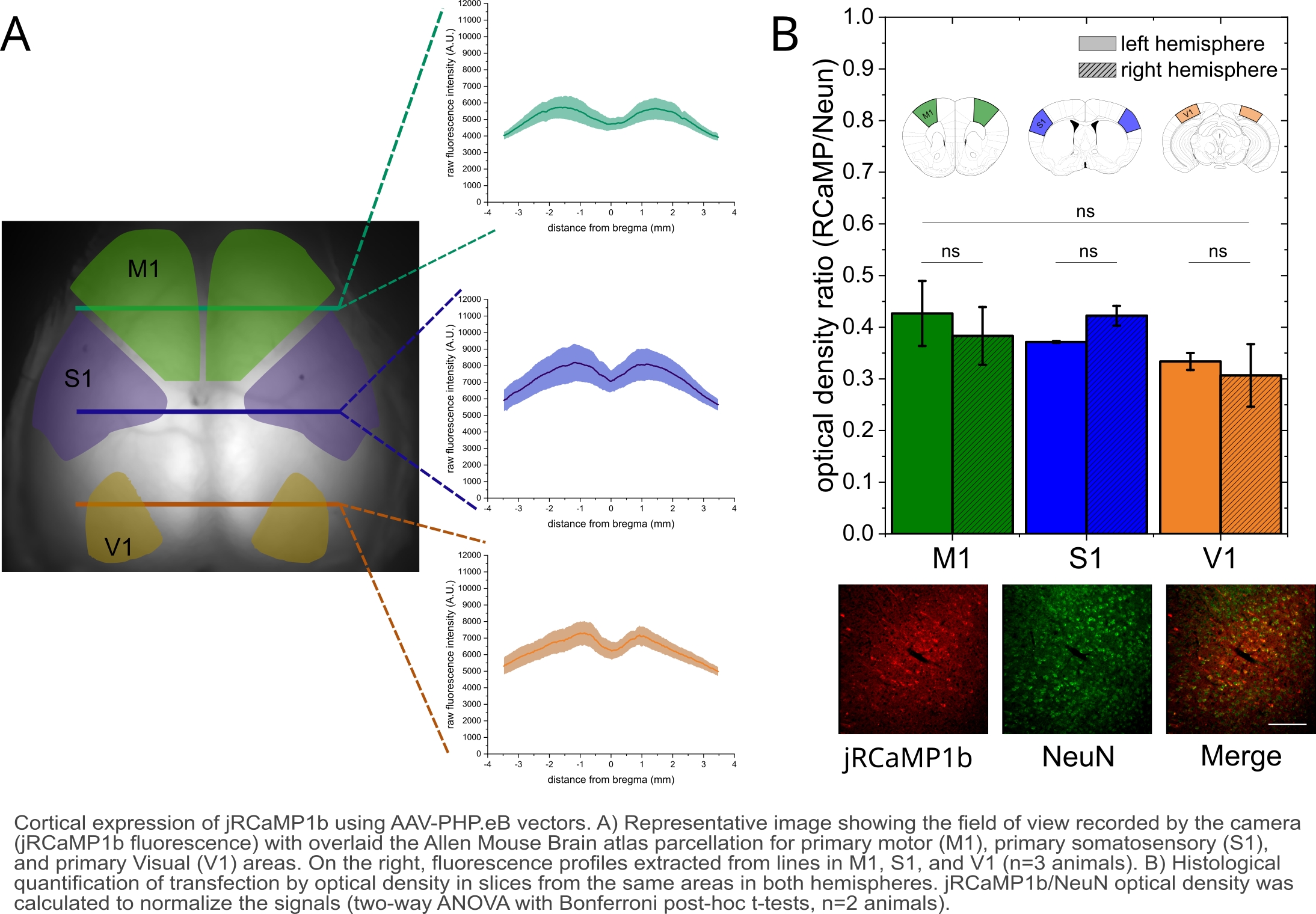

Supplement: Supplementary file 1 [file Image_1.jpg]

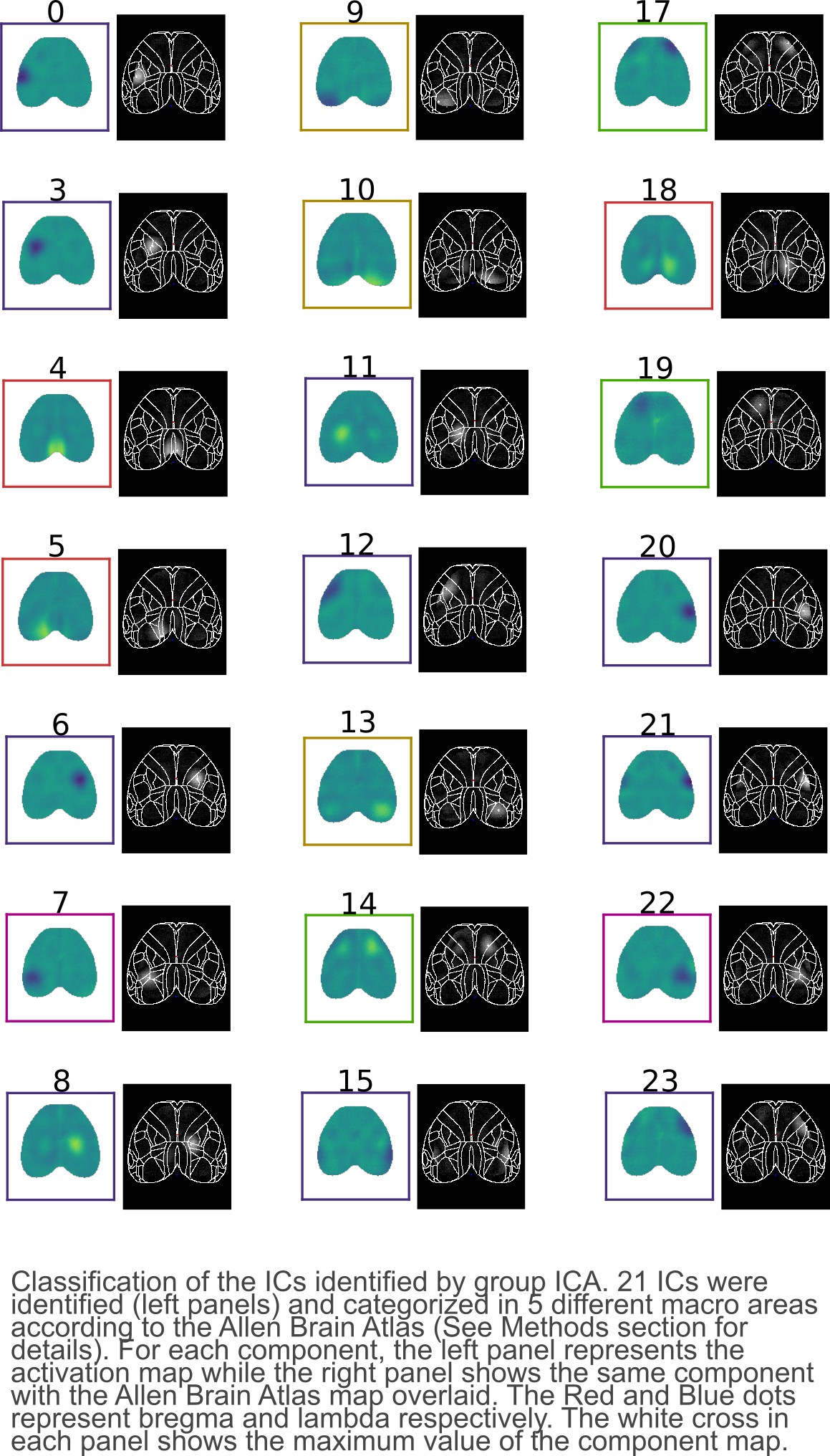

Supplement: Supplementary file 2 [file Image_2.jpg]

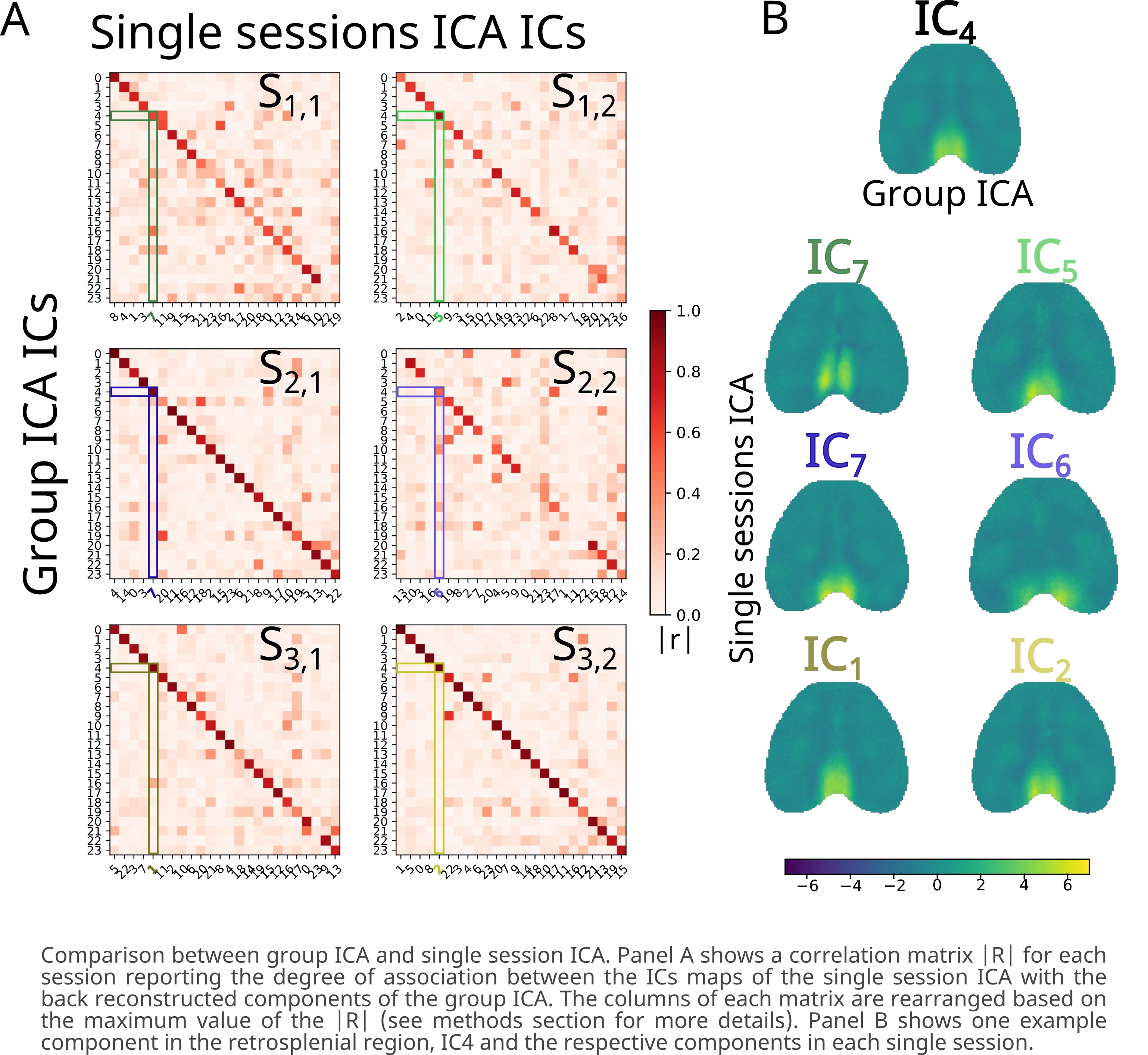

Supplement: Supplementary file 3 [file Image_3.jpg]

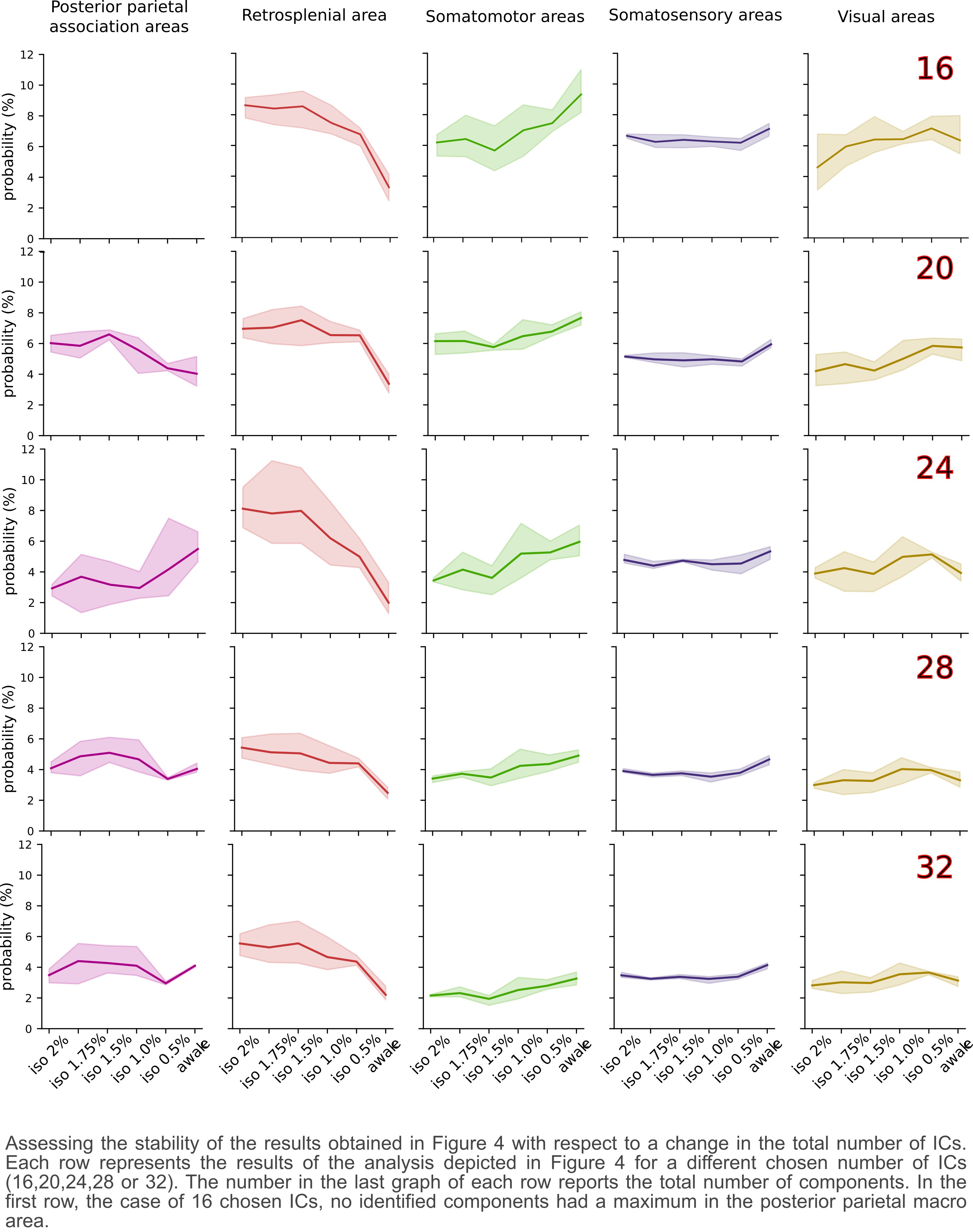

Supplement: Supplementary file 4 [file Image_4.jpg]

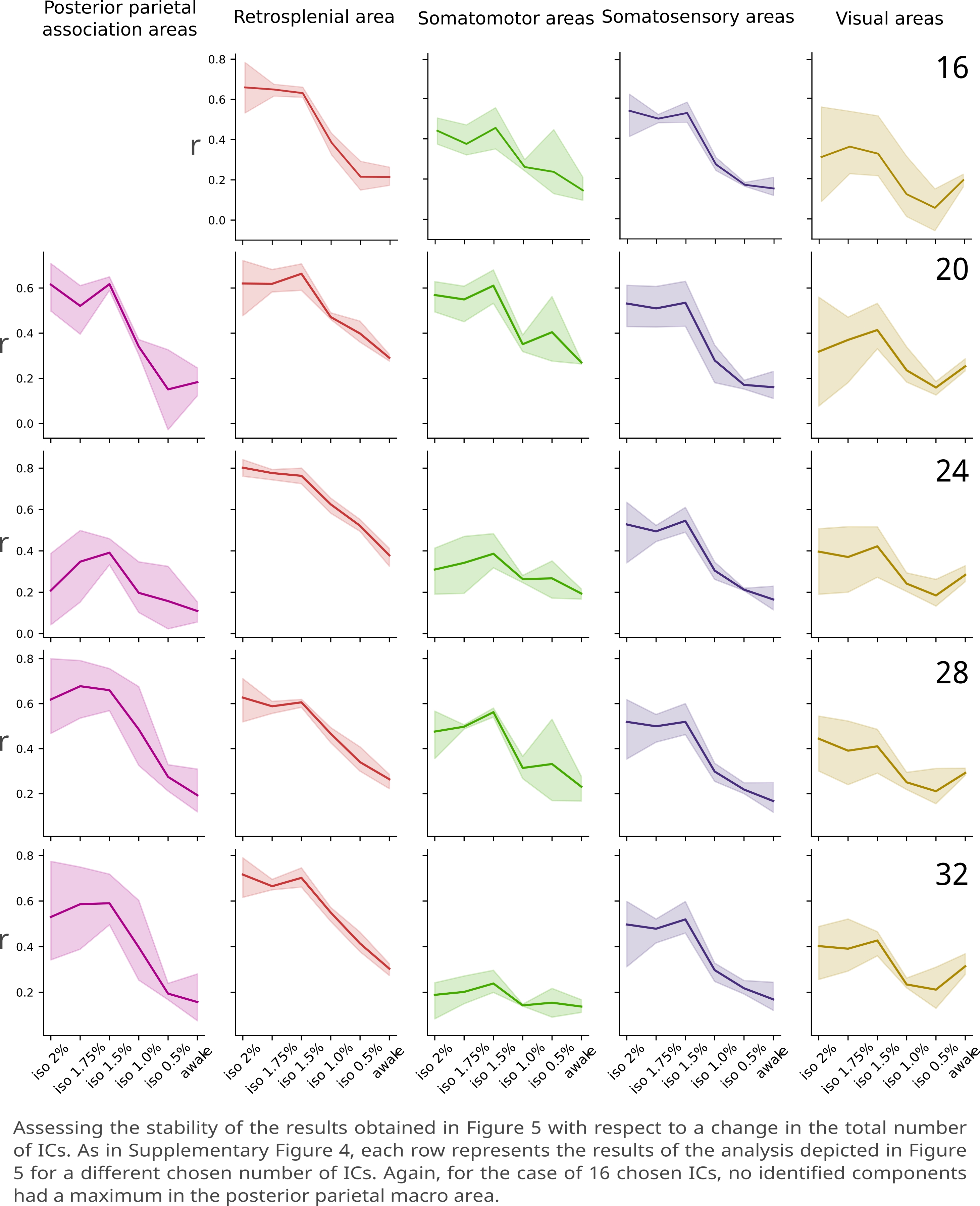

Supplement: Supplementary file 5 [file Image_5.jpg]
